# Validation of the emergency surgery score (ESS) in a UK patient population and comparison with NELA scoring: a retrospective multicentre cohort study

**DOI:** 10.1308/rcsann.2023.0105

**Published:** 2024-03-13

**Authors:** D Clinch, A Dorken-Gallastegi, D Argandykov, A Gebran, JA Proano Zamudio, CS Wong, N Clinch, L Haddow, K Simpson, E Imbert, RJE Skipworth, SJ Moug, HMA Kaafarani, D Damaskos

**Affiliations:** ^1^ Royal Infirmary of Edinburgh, UK; ^2^ Massachusetts General Hospital, USA; ^3^ Royal Alexandra Hospital, UK

**Keywords:** Emergency surgery score, ESS, NELA, Risk prediction

## Abstract

**Introduction:**

Accurate risk scoring in emergency general surgery (EGS) is vital for consent and resource allocation. The emergency surgery score (ESS) has been validated as a reliable preoperative predictor of postoperative outcomes in EGS but has been studied only in the US population. Our primary aim was to perform an external validation study of the ESS in a UK population. Our secondary aim was to compare the accuracy of ESS and National Emergency Laparotomy Audit (NELA) scores.

**Methods:**

We conducted an observational cohort study of adult patients undergoing emergency laparotomy over three years in two UK centres. ESS was calculated retrospectively. NELA scores and all other variables were obtained from the prospectively collected Emergency Laparotomy and Laparoscopic Scottish Audit (ELLSA) database. The primary and secondary outcomes were 30-day mortality and postoperative intensive care unit (ICU) admission, respectively.

**Results:**

A total of 609 patients were included. Median age was 65 years, 52.7% were female, the overall mortality was 9.9% and 23.8% were admitted to ICU. Both ESS and NELA were equally accurate in predicting 30-day mortality (c-statistic=0.78 (95% confidence interval (CI), 0.71–0.85) for ESS and c-statistic=0.83 (95% CI, 0.77–0.88) for NELA, *p*=0.196) and predicting postoperative ICU admission (c-statistic=0.76 (95% CI, 0.71–0.81) for ESS and 0.80 (95% CI, 0.76–0.85) for NELA, *p*=0.092).

**Conclusions:**

In the UK population, ESS and NELA both predict 30-day mortality and ICU admission with no statistically significant difference but with higher c-statistics for NELA score. Both scores have certain advantages, with ESS being validated for a wider range of outcomes.

## Introduction

Emergency general surgery (EGS) admissions accounted for more than 7% of all hospitalisations in the US between 2001 and 2010, with a population-adjusted annual case rate higher than the sum of all new cancer diagnoses.^1^

When compared with elective general surgery as well as nonsurgical admissions, EGS is associated with a significantly higher (up to eightfold) risk of postoperative morbidity and mortality.^2–9^ Emergency surgery remains an independent risk factor for adverse perioperative outcome, even when other variables are adjusted for.^2^ Given these challenges, risk assessment tools have become vital for (1) preoperative counselling of patients and families including informed consent; (2) identification of patients requiring allocation of additional resources such as critical care beds; and (3) standardisation of outcomes between different institutions for quality improvement.^10,11^

Most existing preoperative risk assessment tools were not designed specifically for use in EGS patients and are based on the incorrect assumption that perioperative risk factors have the same impact in elective and emergency surgery.^11,12^ Unlike these tools, the Emergency Surgery Score (ESS) has been developed specifically for use in the EGS population and is not based on this assumption.^13^ It is a point-based score ranging from 0 to 29, derived from four demographic variables, ten comorbidities and nine laboratory data variables.^13^ It has been validated extensively retrospectively and prospectively as an accurate and reliable predictor of postoperative mortality,^10,13^ morbidity and postoperative intensive care unit (ICU) admission.^10,14,15^ The latter is important as EGS patients have longer ICU stays and higher needs for mechanical ventilation and renal replacement therapy,^16^ and outcomes are significantly worse if patients require escalation to a critical care setting compared with direct admission to such a setting.^17^ ESS performs well in the face of missing data elements,^18^ predicts a wide range of outcomes and is superior to ACS-NSQIP in terms of risk prediction in the EGS setting.^19–24^

Except for one study of 102 Greek patients,^25^ this score has been studied exclusively in the US patient population. There are significant differences in the patient population undergoing EGS between the UK and the US as well as important differences in surgical practices and postoperative pathways including ICU admission,^26,27^ which raises questions about the external validity of this score outside the US. For example, the threshold for emergency surgical intervention in the UK is higher than in the US, with a larger proportion of patients being managed nonoperatively and greater associated mortality.^26^ Moreover, there are 2.5-fold more ICU beds per capita in the US compared with the UK, resulting in a much more resource-constrained setting.^28^

National Emergency Laparotomy Audit (NELA) and the Emergency Laparotomy and Laparoscopic Scottish Audit (ELLSA) are national audits aiming to improve the standard of care for EGS patients through collection of high-quality comparative data from all providers of emergency laparotomy in the UK.^29,30^ NELA developed a tool that can be used to calculate an estimated percentage risk of 30-day postoperative mortality in patients undergoing emergency laparotomy.^31^ It is based on 21 variables and has not been validated to predict any outcomes other than mortality. Following recommendations from the NELA audit, patients with a predicted mortality of ≥5% warrant the presence of a consultant surgeon and consultant anaesthetist in the operating theatre as well as postoperative admission to critical care.^29^ Its original derivation was based on a sample of approximately 43,600 patients and an observed c-statistic of 0.86.^31^

The aim of our study was twofold; our first aim was to perform an external validation study of the ESS score in a UK patient population for the prediction of 30-day mortality and postoperative ICU admission. Our second aim was to compare the accuracy of the ESS score with that of the NELA score – the risk prediction score utilised most in the UK.

## Methods

We conducted a retrospective, observational, multicentre cohort study of a prospectively collected group of patients undergoing EGS in the UK. This study was conducted and reported in accordance with the Strengthening the Reporting of Observational studies in Epidemiology (STROBE) standards.

### Patient population

All patients over the age of 18 years undergoing emergency laparotomy between October 2017 and August 2020 at two public tertiary academic centres in the UK (Royal Infirmary of Edinburgh, Scotland and the Royal Alexandra Hospital in Paisley, Scotland) were included. Emergency laparotomy was defined as any laparotomy performed as soon as possible following diagnosis or onset of relevant symptoms where unnecessary delay could potentially endanger the patient's wellbeing and lead to adverse outcome. Emergency trauma, vascular, gynaecological and soft tissue procedures were excluded, as they do not reflect the underlying pathophysiology of the EGS patient population. Laparoscopic procedures (e.g. laparoscopic appendicectomy or cholecystectomy) as well as inguinal hernia repairs were excluded as they do not constitute the same level of risk. For patients undergoing multiple laparotomies during the same admission (e.g. planned re-look procedures or re-laparotomy for complications), only the index procedure was included and patients requiring emergency laparotomy following complications of elective surgery were excluded.

### Data collection and outcomes

All pre-, intra- and postoperative variables (including NELA scores) apart from the ESS score were obtained from a prospective database of all Scottish patients undergoing emergency surgery (ELLSA database). ELLSA is a Scottish Government initiative started in 2017 following a national consensus meeting of all clinical teams with an interest in emergency surgery in Scotland. An agreed minimal dataset is collected prospectively by clinical teams locally in each of the participating acute surgical units in Scotland and uploaded into a dedicated centralised database, where it undergoes a two-stage validation process.^32^

ESS was the only retrospectively derived variable. Data were collected from electronic patient records by doctors in training who were not involved directly in the care of eligible patients. ESS was calculated systematically for each patient based on previously described preoperative variables.^10^ Values at time of decision to operate or the closest possible timepoint were obtained. If a patient had a missing variable, it was treated as the default (i.e. no additional point was assigned). This was based on a previous finding that ESS performs well in predicting outcomes in EGS even when one or more data elements are missing.^18^ As most Scottish laboratories do not routinely collect the serum glutamic-oxaloacetic transaminase (SGOT) levels required in the original score but use alanine aminotransferase (ALT) instead, we adopted a score of 1 for ALT>40U/l following consultation with the original developers of ESS.

Standard American College of Surgeons National Surgical Quality Improvement Program (ACS-NSQIP) definitions were used for all variables.^33^ The primary outcome of this study was 30-day mortality, which was defined as death of any patient within 30 days of index emergency surgery. The secondary outcome of this study was postoperative use of ICU, which was defined as provision of postoperative ICU level of care at any time during the index hospital admission.

NHS National Services Scotland Caldicott Guardian provided ethical approval. Individual patient consent was not required as patient-identifiable information was anonymised before data transmission to the authors. Data were stored and handled in accordance with the EU General Data Protection Regulation and the Data Protection Act 2018.

### Statistical analysis

The agreement between ESS, NELA and each outcome of interest (30-day mortality and ICU admission) was evaluated using the area under the receiver operating characteristic (ROC) curve and c-statistic. The c-statistics of ESS and NELA scores were compared using the DeLong test. The significance level was set at a *p*<0.05. Area under the ROC curve c-statistics (AUC) were classified using Hosmer and Lemeshow's system into poor discrimination for AUC≤0.7, acceptable discrimination for 0.7≤AUC<0.8, excellent discrimination for 0.8≤AUC<0.9 and outstanding discrimination for AUC≥0.9.^34^ The outcomes of mortality and ICU admission were analysed separately. If a patient had a record of one but not both outcomes, they were included in the analysis for which the outcome was recorded and excluded from the analysis for which they were missing the outcome. All data analyses were performed using Stata version 17.0 (StataCorp, College Station, TX).

## Results

**Figure 1:**
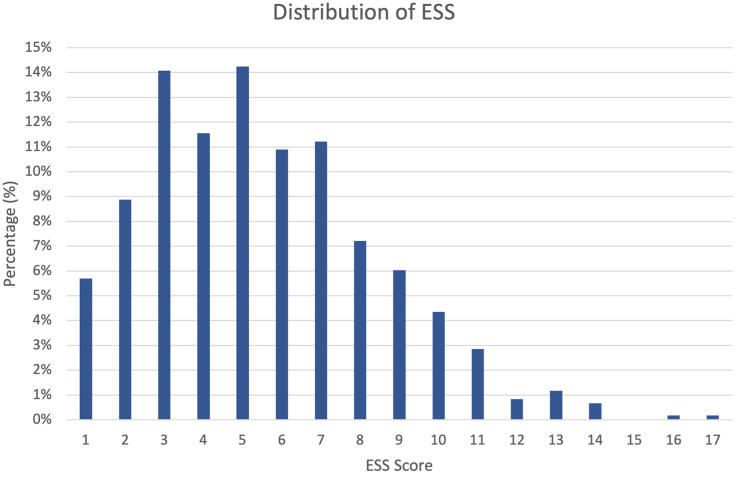
Distribution of ESS in UK patient cohort. ESS = emergency surgery score

**Figure 2:**
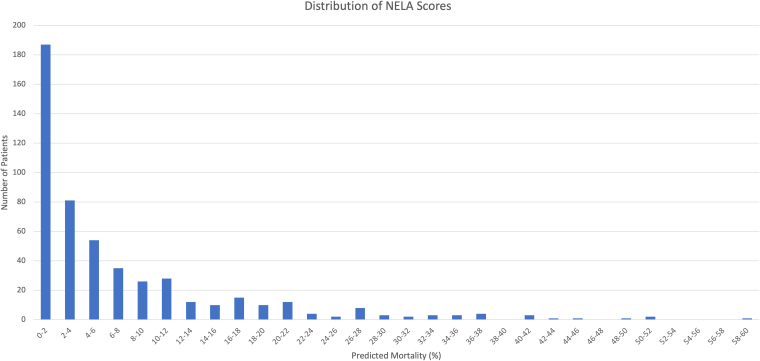
Distribution of NELA scores in UK patient cohort. NELA = National Emergency Laparotomy Audit

A total of 609 patients fulfilled eligibility criteria for this study and were included, of which 387 (63.5%) were treated in Edinburgh and the remainder in Paisley. Median age was 65 years with an interquartile range (IQR) of 52–75 years; 288 patients (47.3%) were male. ESS scores ranged from 1 to 17 points. Median ESS was 5 (IQR 3–7). NELA scores ranged from 0.1% to 59.5%. Median NELA score was 3.7% (IQR 1.1–10). Figure 1 shows the distribution of ESS and Figure 2 shows the distribution of NELA scores in our cohort of patients. Table 1 summarises demographic and surgical characteristics of patients in our cohort. Of the 14,007 variables required to calculate a complete ESS score in 609 patients, there were 956 missing data elements, corresponding to 6.8%. As outlined in Methods, all missing data elements were assigned a score of 0 and included in the analysis.

**Table 1:** Patient characteristics in our cohort

Total number of patients	*N*=609
Median age (years)	65
Male	288 (47.3%)
Female	321 (52.7%)
Operation performed	
Colectomy	159 (26.1%)
Small bowel resection	127 (20.9%)
Adhesiolysis	88 (14.4%)
Peptic ulcer repair	71 (11.7%)
Exploratory laparotomy	49 (8%)
Other	115 (18.9)%

### ESS versus NELA for prediction of 30-day mortality

**Figure 3:**
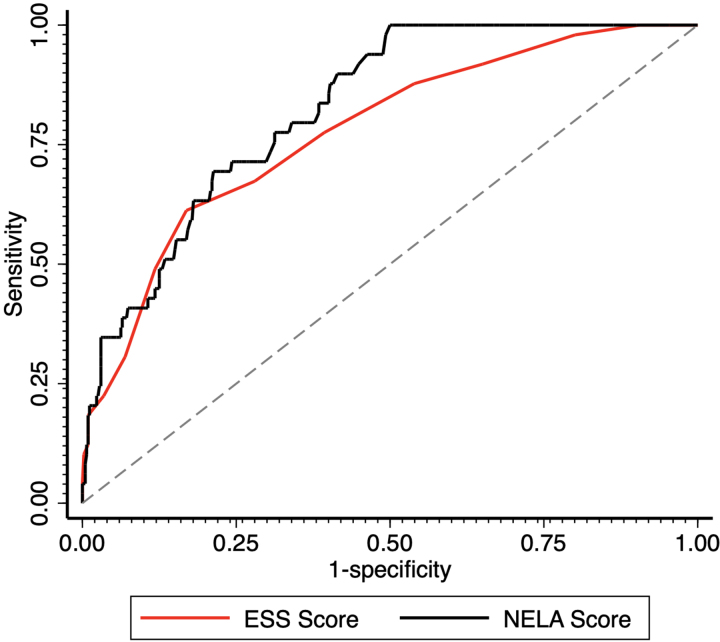
ROC curves for ESS vs 30-day mortality (c-statistic 0.78) and NELA vs 30-day mortality (c-statistic 0.83) in a UK patient cohort. ESS vs NELA for prediction of postoperative use of ICU. ESS = emergency surgery score; ICU = intensive care unit; NELA = National Emergency Laparotomy Audit; ROC = receiver operating characteristic.

A total of 481 patients (79% of the entire cohort) had the outcome measure of 30-day mortality recorded and were therefore included in this analysis; 60 patients (9.9%) died within 30 days of the index operation. As ESS and NELA scores increased, the 30-day mortality gradually increased. Figure 3 shows the ROC curves for ESS and NELA scores and 30-day mortality. Performance of ESS was acceptable, with a c-statistic of 0.78 (95% confidence interval (CI), 0.71–0.85). Performance of NELA score was excellent, with a c-statistic of 0.83 (95% CI, 0.77–0.88). Comparing the two ROC curves for ESS versus NELA scores for prediction of 30-day mortality, we did not identify any significant difference in performance between the two scores (*p*=0.196).

### ESS versus NELA for prediction of ICU need

**Figure 4:**
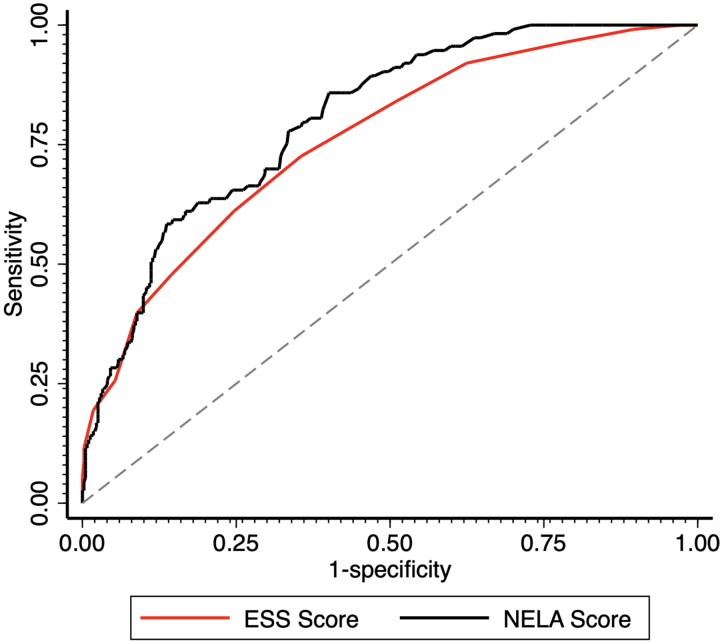
ROC curves for ESS vs need for postoperative ICU admission (c-statistic 0.76) and NELA vs need for postoperative ICU admission (c-statistic 0.80) in UK. ESS = emergency surgery score; ICU = intensive care unit; NELA = National Emergency Laparotomy Audit; ROC = receiver operating characteristic patient cohort.

A total of 145 patients (23.8%) were admitted to ICU postoperatively. A total of 507 patients (83% of the entire cohort) had the outcome measure of postoperative ICU use recorded and were therefore included in this analysis. As ESS and NELA scores increased, postoperative ICU use gradually increased. Figure 4 shows the ROC curves for ESS and NELA and postoperative ICU admission. Performance of ESS was acceptable, with a c-statistic of 0.76 (95% CI, 0.71–0.81). Performance of NELA score was excellent, with a c-statistic of 0.80 (95% CI, 0.76–0.85). Comparing the two ROC curves for ESS vs NELA scores for prediction of ICU admission, we did not identify any significant difference in performance between the two scores (*p*=0.092).

## Discussion

In this study, we demonstrate that ESS is an acceptable predictor of 30-day mortality and postoperative ICU admission in the UK population, thereby fulfilling our first aim of performing an external validation study of this score. The original prospective validation study from the US quotes a c-statistic of 0.84 for mortality and 0.80 for ICU admission,^10^ and our c-statistics of 0.78 for mortality and 0.76 for use of postoperative intensive care are broadly comparable although somewhat lower. Regarding our second aim of comparison of ESS with NELA scoring, our study demonstrates that ESS is not statistically different from NELA when it comes to its ability to predict these outcomes but may be slightly less accurate given the lower AUC values.

There are several limitations to this study. First, we have focused on laparotomies and therefore are not able to make assumptions about other types of EGS procedures, although there is evidence to suggest that ESS is an accurate risk prediction tool for laparoscopic procedures and hernia repairs.^19^ While we used ALT instead of SGOT to perform the ESS score, this can be considered an adequate substitute of underlying liver dysfunction. Collection of outcome data was incomplete, with 30-day mortality outcomes being unavailable in 128 out of 609 patients (21%) and information on ICU need being unavailable in 102 out of 609 (17%) patients. Moreover, ESS scores were derived retrospectively while all other data were held in a prospectively collected database. Morbidity was not evaluated as an outcome in our study as it was not collected routinely in the prospective dataset in a way that was comparable with the US data; whereas the original validation study looked at occurrence of 21 specific postoperative complications that were extracted based on ACS-NSQIP codes, there was no comparable information held in the NELA/ELLSA database, which records information on complication severity but not type. This is a major limitation that will undoubtedly limit the potential impact of this study and poses an important research question for future studies to answer. Finally, we cannot rule out a type II error in the scores performance comparison due to the relatively small study sample size. There may be a true difference in accuracy between ESS and NELA scores that was not detected due to a lack of statistical power, although our c-statistics were comparable with those cited in the literature.^10^

There are several strengths to our study. While calculation of ESS scores was performed retrospectively, NELA scoring and all demographic and outcome variables were extracted from a prospectively collected database. We used inclusion/exclusion criteria, and outcome definitions that were identical to those used in the original prospective validation study.^10^

Comparing ESS with NELA, it remains controversial as to which is the optimal score to use in daily practice. The main advantages of the ESS over NELA scoring include the fact that while NELA has been validated only for the prediction of 30-day mortality, ESS has been validated extensively and shown to be an excellent predictor of multiple outcomes including, but not limited to, morbidity, ICU admission, failure to discharge patients home and unplanned readmission.^14,15,35,36^ ESS has been validated to predict outcomes following a wider range of EGS procedures, including laparoscopic procedures (in particular, appendicectomies and cholecystectomies) as well as hernia repairs,^19^ while NELA focuses only on laparotomies or laparoscopic procedures with bowel involvement. Compared with NELA scoring, ESS does not require the entry of intraoperative variables such as estimated amount of blood loss or degree of intraperitoneal contamination, which are not usually known preoperatively with any degree of certainty, or variables which may be classed as subjective such as American Society of Anesthesiologists (ASA) grade. ESS arguably allows for better quality benchmarking as the intraoperative course could itself be reflective of the quality of operative care provided and not only on patient presenting characteristics.

While most (58.7%) patients in this cohort had NELA scores of 5% or lower, there was a wider distribution of ESS as shown in Figures 1 and 2, indicating that this score may be able to discern finer gradations of risk and therefore have greater discriminatory power. However, its discriminatory power may be limited to its lower range: the highest ESS in our cohort (similar to the original prospective validation study) was 17,^10^ which is associated with a predicted 30-day mortality of approximately 86%, and therefore arguably patients with scores above 20 would not be considered candidates for emergency surgery under most circumstances. It is important to emphasise that while NELA has not been validated to predict ICU admission, a predicted mortality of ≥5% in the UK is commonly used as an indication for postoperative admission to critical care. It is therefore even more notable that ESS was not statistically different from NELA in this context, and previous research has suggested an ESS score of ≥7 as a good cut-off value to indicate need for postoperative critical care.^15^ There is some variation in the literature on ESS with regards to the exact outcome measure used, with some studies using the outcome of ‘ICU need’^15^ whereas others use ‘ICU use’.^10^ Our study employed ‘ICU use’ to be in line with the original prospective validation study. Of note, as there are less than half of ICU beds per capita in the UK compared with the US,^28^ we hypothesise that the vast majority of ICU admissions in the UK will be for absolute clinical indications as the UK is a resource-poorer setting.

In contrast, the main advantage of the NELA score over ESS relates to the fact that a percentage mortality estimate is more intuitively meaningful and more easily understood by patients and doctors when compared with a numeric value that requires context for its interpretation. Ultimately, further research is required to assess the impact of these scoring systems on decision making in EGS patients in the real-world setting.

While assessment tools such as NELA score and ESS assume a linear and cumulative impact of risk factors, there have been advances in the development of interactive, nonlinear risk calculators using machine-learning techniques with the potential of continuously improved accuracy through ongoing learning by artificial intelligence.^37,38^ The Predictive OpTimal Trees in Emergency Surgery (POTTER) calculator has been developed as such a tool for use in EGS patients and uses the machine learning technique of Optimal Classification Trees.^37,39^ It has been shown to have a higher mortality c-statistic than ESS, with the number of questions to predict mortality ranging from 4 to 11.^37^ Such techniques are currently still under development and may improve our ability to predict outcomes in EGS patients beyond ESS in the future.

How can ESS help the clinician in everyday practice? We recommend that ESS should be calculated for all EGS patients admitted to hospital for whom surgery is considered as part of the management plan. Patients with scores ≥5 (which correspond to a 30-day mortality of 5.8%) should be considered ‘medium risk’ and patients with scores ≥7 (which correspond to a 30-day mortality of 13.2%) should be considered ‘high risk’, which can inform discussions around consent with patients and families.^10^ In terms of ICU admission, scores ≥7 should prompt a discussion with the critical care team as postoperative admission may be warranted.^15^ Similar cut-off values can be found for all other outcomes for which ESS is validated and can guide other aspects of postoperative care. Finally, data on ESS scores should be stored as part of the national audit dataset to allow for fair comparison between outcomes of different units that have been adjusted for surgical risk.

One should bear in mind that it is not appropriate to withhold any treatment, including surgery, based on a risk score alone.^40^ Twenty percent of patients undergoing emergency laparotomy in the UK have a P-POSSUM predicted 30-day mortality greater than 25% and it is important to recognise that a small proportion of patients can do very well despite high predicted perioperative risk.^40^ Therefore, the current recommendation by the Royal College of Surgeons of England is that patients who have a high predicted perioperative mortality risk should be assessed preoperatively in person by a multidisciplinary team including a consultant surgeon, anaesthetist, intensivist and palliative care specialist.^40^ In this context, risk prediction tools are useful in triggering this input as well as being part of the global assessment of a patient, informing discussions with patients and relatives, and resource allocation.

In this study, we demonstrate that ESS is an acceptable predictor of 30-day mortality and postoperative ICU admission in the UK population, and that ESS is not statistically different to NELA when it comes to its ability to predict these outcomes.
